# Gastric Neuroses

**Published:** 1912-08

**Authors:** William J. Mallory

**Affiliations:** Washington, D. C.; Instructor in Medicine in the George Washington University and Attending Physician to the Out-Patient Department of the University Hospital


### GASTRIC NEUROSES.

By William J. Mallory, M. D., Washington, D. C.
*Instructor in Medicine in the George Washington University and Attending Physician to the Out-Patient Department of the University Hospital.*

Under the above title it is usual to consider all those disturbances of digestion not associated with any demonstrable lesion. It is true that some of the diseases formerly considered functional have since been found to be accompanied by anatomical changes (e. g., hyperchlorhydria), and it is probable that additional knowledge will cause a revision of the old classification, but for the present the well-known distinction must suffice.

Much that follows may very well apply to intestinal neuroses also, for it is rare to find a disturbance of one part of the digestive tract without other portions being more or less involved. This is especially true of the functional disturbances.

The classification most widely accepted divides the gastric neuroses into: 1. Secretory, 2. Sensory; and 3. Motor disturbances, and each of these may be either irritative or depressive. They may also be divided into monosymptomatic, and polysymptomatic varieties.

This classification is adequate in that it provides for every variety of disturbance, but it bears no relation to etiology, and the terms used have this disadvantage, they tend to keep attention focused upon the stomach, when, as a matter of fact, the more there is learned about the factors controlling the function of the organs of digestion, the less probability there seems to be that there is such an entity as a primary gastric neurosis, having its seat in the nerves of the stomach.

Reflex disturbances due to disease of other organs should not be considered in this connection, for they are not neuroses, but symptoms due to organic lesions elsewhere, for example, appendicitis, cholelithiasis, etc. Most disorders of digestion not due to reflex irritation are either neuroses with gastro-intestinal symptoms or manifestation of some definite nervous disease.

The differentiation of these groups, however, is difficult, and diagnosis is only possible after the most careful study.

Here the various physiological tests, including chemical examination of secretions and excretions, are of great value, but those who have used them most, and checked the findings at operation or post-mortem know their limitations best.

Frequently, after exhausting all such means of investigation, it is necessary to again review the family history, personal history, and more especially to inquire with the greatest detail into the personal habits, social and family relations of the patient in order to find the real cause of the disturbance, and even then it is sometimes necessary to observe the patient for a while before making a definite diagnosis. Having excluded reflex irritation, and organic disease of the gastro-intestinal tract, it must then be determined whether the disturbance is a symptom of organic nervous disease or a neurosis. The relation of locomotor ataxia to gastric disturbance is too well known to require more than mention, but the following case illustrates that epilepsy must not be forgotten, especially when dealing with children.

Case No. 1.—H. S., female, aged six and a half years.

At the age of two years she fell backward from a high chair, apparently without any serious consequences.

Six months later she is said to have had "indigestion and convulsions." She would place her hand on the abdomen over the epigastric region, and cry, "Mama, I've got a pain, take me quick." She would then run to her mother and have a convulsion, remain unconscious for a few minutes, and then sleep from a half to several hours. On some occasions she remained unconscious as long as twelve to thirty-six hours, and her general condition was such that the attending physician considered tubercular meningitis. After this illness she remained well till her sxth year.

For the past six months she had been having the above-described painful attacks and convulsions with varying frequency, sometimes as many as five in one day and again remain free from them as long as nine days. Her mother has given her high salt enemata several times a week and thinks she is always better after these or a purgative.

The diet has consisted of milk, broths, cereals, milk toast, with salt no it. The mother states that after an enema or laxative the child passes a great deal of mucous, and that after an attack there is aructation of much gas, the breath is foul, and curds are sometimes vomited with much relief.

On her first visit to the office she had an attack very much as her mother had described. She called out in a frightened tone, was taken on her mother's lap, the eyes rolled up and to the left, the legs and thighs were flexed on the abdomen, the left hand and fore-arm on the chest. In a few seconds she relaxed, and when placed on a couch, remained there in a stupid condition a few minutes, then got up and said she was hungry, and began to play as if quite well.

The physical examination was absolutely negative.

Repeated examination of the blood, urine and feces made on several different occasions revealed nothing abnormal.

A complete examination of the nervous system by a neurologist disclosed no sign of any lesion whatever.

About four months later death occurred from typhoid fever (Widal positive), complicated by pneumonia. On post-mortem no lesion of the brain was found, and the gastro-intestinal tract was free except a congestion of the lower part of the ileum.

In considering this case it is to be remembered that epilepsy occurs more frequently in the first and second years than in any other two except the fourteenth and fifteenth, that sensory aurae are the most common and the gastric the most common of these. The attacks are frequently followed by eructations of large quantities of gas, and an exceedingly foul breath. Over-eating, indigestion, and constipation are among the common causes precipitating a seizure.

Most cases roughly diagnosed as "nervous indigestion" are true neuroses, with gastro-intestinal symptoms and should be treated as such. They are best considered in the light of Freud's illuminating contribution on the anxiety neuroses, which he has carved out of that too heterogeneous mixture, neurasthenia.

The anxiety neurosis, according to Freud, has as its ever present cardinal symptom, first, a general increased irritability, shown by increased reaction to all impressions, including those received through the special senses, especially the ear. Second, in addition to this, there is a condition of anxious expectation. For example, if a pattent goes walking it is in fear of the weather, a slight suggests some serious disease, or if a member of the family is late returning home they must have met with some serious accident. Third, the anxiety attack. This may occur suddenly or develop gradually, and is described as follows: The patient has the feeling as if death were near, or he is about to have a "stroke." The heart seems to stand still, something stops the breath, there is pallor, dizziness, chilliness, perspiration, palpitation, a desire to urinate and sometimes diarrhoea and tenesmus.

There are also a number of minor disturbances which may occur alone or in association with each other. These are known as "equivalents."

(a) Disturbances of the heart's function, i. e., palpitation, tachycardia, arythmia, and pseudo angina pectoris.

(b) Disturbance of breathing, such as asthmatic attacks.

(4) Incontrollable shaking and trembling.

(e) Bulemia and vertigo.

(f) Diarrhoea.

(g) Incordination and ataxia.

(h) Congestions or the so-called vasomotor neurasthenia.

(i) Paraesthesiae.

(j) Sudden awakening with fright; fear of falling.

(k) Urgent desire to urinate.

(l) Muscular cramps.

Freud considers the anxiety neurosis nearly always due to some damaging irregularity in the sexual life which results in a dislocation of the psychic from the somatic stimulus of the sexual act.

Stekel seeks the cause in some psychic conflict, for example, when under the influence of two conflicting desires, the weaker one, when suppressed and often forgotten, will manifest itself as an anxiety which having become disassociated from its original object (free floating anxiety) then readily becomes associated with any object or circumstance which may seem to be a logical cause for anxiety. For example, a person having such a free-floating anxiety may notice some gastric, cardiac, or intestinal disturbance and assume that one of these organs is diseased and is the cause of all the discomfort and mental perturbation.

The followng case will serve as a good example of such a condition:

Case No. 2. Mr. R., male, aged 47, law clerk, married and has one child.

Father died of tuberculosis at age of 45. Mother is said to be nervous and is in an institution for mental disesases. Patient has one brother, who is living, in good health. He has had many children's diseases but no scarlet fever, diphtheria, or rheumatic fever. Leads a sedentary life, uses alcohol moderately and regularly, and smoked excessively till recently. Eats a moderately full, mixed diet.

He complains of attacks of indigestion and heart trouble. These began about five years ago, and are described as a sinking sensation in the epigastrium, which gradually "spreads to the head and whole body," followed by the eructation of gas. The attacks most frequently occur after he has retired and is just falling asleep, or early in the morning before he arises.

On the occasion of the last attack he awoke with a start, feeling that "something terrible" was about to happen; he felt a sinking sensation in the stomach, weakness in the legs, and palpitation of the heart.

On these occasions he must get out of bed and read for hours before he can again go to sleep. He has been told that he has a heart lesions, and has been taking strophanus nitroglycerine and sodium bromid. He is a small, poorly nourished man of ashy grey complexion, with some puffiness under the eyes. The lungs are intact except a mild bronchitis. There is a systolic blowing musical heart-murmur, heard best over the apex and transmitted into the axilla, and an alternating pulse.

An examination of the sputum, blood, urine, feces and gastric contents revealed no sign of any organic disease. Digestion, absorption and elimination seemed quite normal. He complained of no pain, could eat a meal of beef, steak, fried potatoes and vegetables without discomfort, but would not leave the house to go to work, or the physician's office for fear of "an attack" while in the car or on the street.

In this case, the compensated mitral lesion was not considered aquate to account for the symptoms, and further inquiry into the history and personal habits was made, because the neuropathic history, the nature of the symptoms, the time of their appearance, and the condition of well-being between the attacks suggested a neurosis, based upon or related to the sexual life. On inquiry the patient acknowledged that he had practiced coitus interruptus for several years.

This practice is a frequent cause of neuroses. The harmful effects are due not only to the interruption of the act before completion, which in itself is enough to disturb the nervous equilibrium, but because it is performed in a state of anxiety and fear, for one reason or another. The anxety is repressed, an attempt made to dismiss it from the mind, it becoms disassociated from its original object and later manifest itself in full force in association with the first object or circumstance which seems to be an adequate cause for such a feeling, which in this particular case was a
cause for such a feeling, which in this particular case was a

The attacks naturally occurred most frequently just as he was falling asleep, or early in the morning before awakening, because at these times the higher centers of control were relaxed and the suppressed anxiety return to consciousnss.

The patient was advised with regard to his habits, heart stimulants were discontinued, a harmless diet ordered, a tonic precribed, and he was given a pungent sedative for the sinking sensation in the stomach. Since then he had had one slight attack, and now, five months later, is well.

Psychic vomiting may be profitably considered in connection with the neuroses, for rational treatment of both is dependent upon an understanding of the manner in which they are produced.

The feeling of disgust or shame, so closely associated with nausea is not natural, but acquired, and is developed by education and training. An infant has no sense of digust or shame. Before it is taught otherwise it will put any and everything into its mouth. It must be taught that certain things are unclean or harmful, just as it must be taught modesty, and in order to accomplish this it is taught to have a feeling of revulsion toward certain objects, and a sense of shame in association with certain acts. Thus begin self control, inhibition, and repression.

The reflexes of sucking, deglutition, defecation and micturation are among the earliest to be developed. The first bodily sensations of comfort, well being and satisfaction are associated with the normal performance of these vegetative functions, and the reception of agreeable sensations from the erogenous zones. Later, when the sexual sense develops, it is usually localized in one area, and to a great extent disassociated from the mere primitive erogenous zones, but never entirely so, and sometimes scarcely at all, as seen in certain perverts. Therefore, when desire, disgust, revulsion and shame are awakened, and inhibition and repression are exercised, they act not only on one, but more or less on all the functions with which they were originally developed and associated, and in this manner produce symptoms which at first do not at all suggest either the location or nature of the disturbance.

Psychic shock, mental conflict, anxiety, repression and inhibition, are correctly given as causes of psychic vomiting, but unless the cause of these emotions can be discovered, this knowledge is of very little practical value. In civilized life it is preeminently in the sexual domain that these emotional dsturbances occur, and according to Freud it is here that we much search for the cause of many cases of psychic vomiting.

The following case of regular, periodic vomiting seems interesting enough to bring to your attention, although it is not analyzed as completely as it should be and does not seem to be dependent upon any emotion related to the sexual appetite:

Mr. J., aged 31, occupation, motorman.

*Family History.*—Father was a man of intelligence and ability, having been a train dispatcher, but was addicted to the excessive use of alcohol, and died while intoxicated.

Mother died of womb disease while patient was an infant.

Sister living.

The patient had measles at age of ten, and typhoid at age of fifteen. From that time till about four years ago when he noticed that he became "nervous" on going into a crowd.

*Present Illness.*—Can not say when stomach trouble began, because he always had "a weak stomach." At present he complains of nausea and vomiting, which occurs every morning while he is walking to the car barn to take out the car for the first run at six o'clock.

When he is in the car collecting fares he becomes nauseated, the knees feel weak and tremble, there is a desire to go to stool and to expectorate.

The nausea never occurs at other times of the day, after other meals, appetite is excellent and he eats all kinds of food, but in rather restricted quantities. Patient sleeps well.

On account of the nature of his work, the patient's habits are very irregular. He usually rises at about four-thirty in the morning, gets a breakfast of eggs, bread and coffee, walks to the car barn, works from six to ten o'clock, then returns home and rests until two-thiry, when he returns to the car barn to take his car out at four o'clock and work till one o'clock in the morning. Careful questioning revealed no bad habits of any kind.

*Physical Examination.*—A very small, undersized, thin man of poorly developed muscleature and poor nutrition. Habitus Aesthenicus, i. e., a long, narrow, flat thorax with acute costal angle. Abdominal muscles thin and relaxed, lower portion of abdomen prominent. Kidneys not palpable, stomach not below the umbilicus.

The patient's general appearance was what is usually called neurasthenic. An examination of the digestive functions revealed nothing more than a slight subacidity, and a tendency to constipation. In fact, nothing sufficient to account for his symptoms, especially their peculiar regularity.

A diagnosis of inanition, neurasthenia and dyspepsia with sub-acidity would cover the case, and suggest a suitable line of treatment, but it would leave one in the dark as to the cause of the claustophobia and anthrophobia, the peculiar time and place of the nausea and vomiting, and would not help toward etiological treatment.

A provisional diagnosis of an anxiety neurosis was made and an attempt begun to find its origin.

A more detailed inquiry into the life, work, habits, etc., of the patient just prior to the onset of these symptom,s revealed that he had been a flagman on the railway, and one night while helping to shift some cars to a lonely siding he had found a decapitated human body, which had apparently been run over by the train and left on the side of the track. He related with great fluency and detail that he was sometime alone with this body, before any one came to his assistance, and while waiting he heard some one run under the train as if sneaking away. He believed that the man had been murdered and placed on the track afterwards in order to conceal the fact.

After this time he was always afraid of finding bodies on the track and could always see mangled forms when he went by this an other lonely places. On another occasion he did find an injured man by the railway and was compelled to support him in his arms on top of the train till the dying man was brought to the next station.

Prior to making this detail inquiry the patient had told me that he was *not* afraid of the vacant lot near the car barn, although people had been sandbagged there and robbed.

It was soon after this experience on the railway that the patient noticed that he was nervous in crowds.

It will be noted that the patient voluntarily stated that he was not afraid to pass the lonely vacant lot on the way to his work early in the morning while it was still dark, although he believed that more than one person had been sandbagged there and robbed.

It seems that since his shocking experience with the body of a man whom he believed to have been murdered, he dreaded lonely places, especially in the dark, and was doubtless really much afraid in passing this spot in the dark morning hours, but by exercise of the will he supressed the emotion of fear. The conflict was sufficient to produce nausea and vomiting, the fear anxiety having been suppressed became free-floating and attached itself to the next most obvious annoying circumstance, that was the to him disagreeable work of collecting fares in a crowd of toughs, the anxiety then expressed itself in the usual manner, i. e., weakness, trembling, a desire to go to stool, to urinate and expectorate. It is well known that agoraphobia and claustrophobia frequently alternate with each other.

A detailed discussion of the treatment of gastric neuroses would require more space than is here available, but a brief mention of the more important principles should not be omitted.

In the first place it must be remembered that the existence of a neurosis does not exclude organic disease. Both may be present without bearing a causal relation to each other.

Therefore, previous to beginning treatment of the neurosis, every care should be exercised to recognize co-existing organic disease, and if this be present it should receive suitable treatment.

In the absence of organic disease, the common practice of giving tonics or sedatives, and a rest cur,e may be futile work, unless these measures be applied ina new environment and under changed circumstances, that is, with the patient removed from the place, mode of life, person, or object in association with which the mental conflict has developed. Herein lies the secret of many cures made at watring places, or after a journey to a different climate, and it is also the reason why so many relapses occur when the patient it brought back to the old scene of the disturbing cause.

If the patient is intelligent, and the neurosis a simple one of recent development, and if the cause can be discovered, it is sometimes sufficient to explain the nature of the condition to the patient, and give the necssary directions with regard to correcting vicious habts, and making the required changes n the environment, meanwhile treating existing symptoms in a rational manner.

Some cases are more obscure, and require a careful and complete psychoanalysis before the cause of the neurosis can be discovered. This is especially rue of cases of long standing, in which the memory of the circumstances which gave rise to the mental conflict and anxiety neurosis may have been repressed for so long that they can not be recalled to memory voluntarily. Such cases, after a preliminary examination should be recognized for what they are, and referred to a competent psychiatrist, or the result may be very disastrous.

From what has preceded it is hoped that it is apparent how vain and futile it is to make a diagnosis of nervous indigestion, nervous diarrhoea, or psychic vomiting, and treat the patient with tonics or sedatives without attempting to determine the exciting cause, for n his class of diseases more than any other successful therapy must rest upon etiology.

1720 Conn. Ave., N. W.

---

The removal of one kidney without a definite knowledge of its mate's function is one of surgery's most unscientific procedures.

If a vesical growth is but even faintly suspicious of malignancy let your incision include all coats of the vesicus.

A few minutes spent in reading the pathological anatomy of chronic urethral inflammations will help you materially in finding an explanation of the continuance of a discharge which persists in spite of injections.—*American Journal of Dermatology.*

